# Analysis of *Camellia oleifera* transcriptome reveals key pathways and hub genes involved during different photoperiods

**DOI:** 10.1186/s12870-022-03798-0

**Published:** 2022-09-12

**Authors:** Jindong Yan, Jiacheng He, Jian’an Li, Shuangshuang Ren, Ying Wang, Junqin Zhou, Xiaofeng Tan

**Affiliations:** 1 Key Laboratory of Cultivation and Protection for Non-wood Forest Trees of Ministry of Education and the Key Laboratory of Non-Wood Forest Products of Forestry Ministry, Central South University of Forestry and Technology, 410004 Changsha, China; 2 Engineering Technology Research Center of Southern Hilly and Mountainous Ecological Non-Wood Forest Industry of Hunan Province, 410004 Changsha, China

**Keywords:** *Camellia oleifera*, Transcriptome, Photoperiod, Regulatory pathways, Hub genes

## Abstract

**Background:**

*Camellia oleifera* Abel. (*C. oleifera*) is an important traditional woody species in China that produces edible oil. However, the current literature lacks a proper understanding of *C. oleifera*’s ability to adapt to different photoperiods.

**Results:**

Our results indicate that the photoperiod can significantly impact flowering time in *C. oleifera*. We grew a total of nine samples under the short day condition (SD), middle day condition (MD) and long day condition (LD). Transcriptome analysis yielded 66.94 Gb of high-quality clean reads, with an average of over 6.73 Gb of reads for per sample. Following assembly, a total of 120,080 transcripts were obtained and 94,979 unigenes annotated. A total of 3475 differentially expressed genes (DEGs) were identified between the SD_MD, SD_LD, and MD_LD gene sets. Moreover, WGCNA identified ten gene modules. Genes in pink module (92 genes) were positively correlated with SD, and negatively correlated with both MD and LD. Genes in the magenta module (42 genes) were positively correlated with MD and negatively correlated with both LD and SD. Finally, genes in the yellow module (1758 genes) were positively correlated with both SD and MD, but negatively correlated with LD. KEGG enrichment analysis revealed that genes in the pink, magenta, and yellow modules were involved in flavonoid biosynthesis, amino sugar and nucleotide sugar metabolism and circadian rhythm pathways. Additionally, eight hub genes (*GI*, *AP2*, *WRKY65*, *SCR*, *SHR*, *PHR1*, *ERF106*, and *SCL3*) were obtained through network analysis. The hub genes had high connectivity with other photoperiod-sensitive DEGs. The expression levels of hub genes were verified by qRT-PCR analysis.

**Conclusion:**

An increase in light duration promotes earlier flowering of *C. oleifera*. Flavonoid biosynthesis, amino sugar and nucleotide sugar metabolism, and circadian rhythm pathways may function in the photoperiodic flowering pathway of *C. oleifera*. We also identified eight hub genes that may play a role in this pathway. Ultimately, this work contributes to our understanding of the photoperiodic flowering pathway of *C. oleifera* and further informs molecular breeding programs on the plant’s photoperiodic sensitivity.

**Supplementary Information:**

The online version contains supplementary material available at 10.1186/s12870-022-03798-0.

## Introduction

*Camellia oleifera* Abel. (*C. oleifera*), belongs to the tea family (Theaceae) [1], and is one of the four major woody oil plants in the world. The oil produced by *C. oleifera*, known as “eastern olive oil”, has suitable nutritional value and health benefits – it can reduce serum triglycerides, has good stability against oxidation and increases the body’s level of high-density lipoproteins [2, 3]. In addition to cooking, *C. oleifera* oil is extensively used in traditional Chinese medicine, as well as for manufacturing soap, margarine, hair-oil, lubricants, and rustproof oil [4–6]. *C. oleifera* is widely distributed throughout the subtropical mountainous areas of South China, especially in Jiangxi, Hunan, and Guangxi provinces [7]. With the rapid development of the *C. oleifera* industry, there is a clear need for the cultivation of *C. oleifera* varieties in new areas. As part of their effective cultivation, it is vital that varieties of *C. oleifera* blossom and bear fruit at the proper time.

Exotic plants can be influenced by variety of factors, such as temperature, humidity illumination and seasonal changes in day length (photoperiods) [8]. The ability of plants to adapt to different photoperiods is crucial for successful exotic introduction. Plants have a series of response systems that enable accurate and timely photoperiod adaptation [9]. In *Arabidopsis*, a series of major proteins, encoded by *PHYA/PHYB*, *CRY1/CRY2*, *GI*, *FKF1/ZTL/LKP2*, *CCA1/TOC1/LHY*, and *CDFs* are involved in this environmental cue [10–15]. Together, these proteins act to induce the expression of *CO*, a key photoperiod transcription factor [16, 17]. CO protein can directly regulate the expression of *FT*, which plays an important downstream target in many regulatory plant flowering pathways. Moreover, the FT protein can move from the phloem cells of a leaf toward its apex to activate the floral integrator genes *AGL20*, *LFY* and *AP1* [18–24]. However, both the photoperiodic sensitivity and flowering mechanism of *C. oleifera* remain uncharacterized.

To explore the photoperiod response mechanism of *C. oleifera*, we determined the flowering time of the *C. oleifera* ‘Huashuo’ grown under a short day condition (SD: 8 h light/16 h dark), medium day condition (MD: 12 h light/12 h dark), and long day condition (LD: 16 h light/ 8 h dark). Then, we performed global RNA sequencing (RNA-seq) analysis to investigate the underlying biological mechanisms that correspond to different photoperiod conditions. We also used quantitative real-time RT-PCR (qRT-PCR) to detect the expression of hub-genes. Overall, this study enhances our understanding of the flowering mechanisms that operate during different photoperiods in *C. oleifera*.

## Results

### *C. oleifera* phenotype and physiological index by photoperiod

**Fig. 1:**
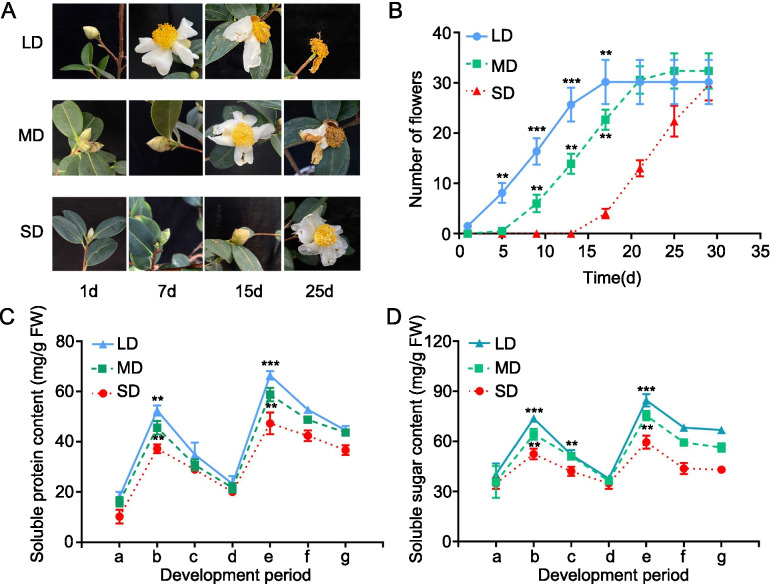
Effect of different photoperiod treatments on flower time, soluble protein content and soluble sugar contents in *C. oleifera* ‘Huanshuo’ leaves. **A** Represent image of *C. oleifera* flower in SD, MD, LD conditions. **B** The number of *C. oleifera* flower in SD, MD, LD conditions. Soluble protein content (**C**) and soluble sugar content (**D**) of *C. oleifera* in SD, MD, LD conditions. a-g represent seedling stage (a), flower bud differentiation (b), budding (c), bud breaking (d), pre-flowering (e), initial flowering (f) and full blooming stage (g) respectively. The first bud bloom time of all treatments set as 1d. Significant differences are indicated: **P < 0.01, ***P < 0.001 (Tukey’s least significant difference test)

To explore the sensitivity of *C. oleifera* to different photoperiods, we determined the flowering time of *C. oleifera* grown in the SD, MD, and LD conditions. We found that the flowering time of *C. oleifera* grown in the LD condition was significantly earlier than those in the MD and SD conditions, while the flowering time of *C. oleifera* grown in the MD condition was significantly earlier than that in the SD condition (Fig. 1A, B). Consistent with the flowering phenotypes, the soluble protein and soluble sugar contents of *C. oleifera* grown in the LD condition were also higher than those in the MD and SD conditions, while the soluble protein and soluble sugar contents of *C. oleifera* grown in the MD condition were significantly higher than that in the SD condition (Fig. 1C, D). These results suggest that the duration of light significantly impacts flowering time in *C. oleifera*.

### Characterization of the transcriptome in the leaf of *C. oleifera*

To explore the mechanism causing the different flowering time of *C. oleifera* grown under the SD, MD, and LD conditions, we conducted the transcriptomic analysis of the *C. oleifera* leaf. Table S1 summarizes the RNA-Seq data derived from our nine cDNA libraries (SD_1, SD_2, SD_3, MD_1, MD_2, MD_3, LD_1, LD_2, and LD_3). After quality control, we obtained 66.94 Gb of high-quality clean reads, with each sample containing an average of more than 6.73 Gb. Additionally, the Q30 base content of each sample was greater than 93.25%. The proportion of mapped reads per library ranged from 81.66 to 92.17%. A total of 120,080 transcripts were obtained from all clean reads. There were 199,518 (99.59%) unigenes with a length greater than 200 bp and 51,877 (25.89%) unigenes with a length greater than 1800 bp. The length distribution of the transcripts is shown in Fig. S1.

Our set of 94,979 non-redundant high-quality unigenes was annotated by searching against common functionality databases. Among our set, 79,973 (84.2%), 41,182 (43.36%), 86,329 (90.89%), 94,504 (99.5%), 76,455 (80.5%), and 80,761 (85.03%) annotated unigenes were obtained from the GO, KEGG, COG, NR, Swiss-Prot, and Pfam databases, respectively (Fig. S2). PCA of the nine samples suggested that the variation between biological repetitions conformed to the expectations of experimental design (Fig. S3). These results supported the use of our transcriptome data for subsequent analysis.

### Functional analysis of DEGs in different photoperiods

**Fig. 2:**
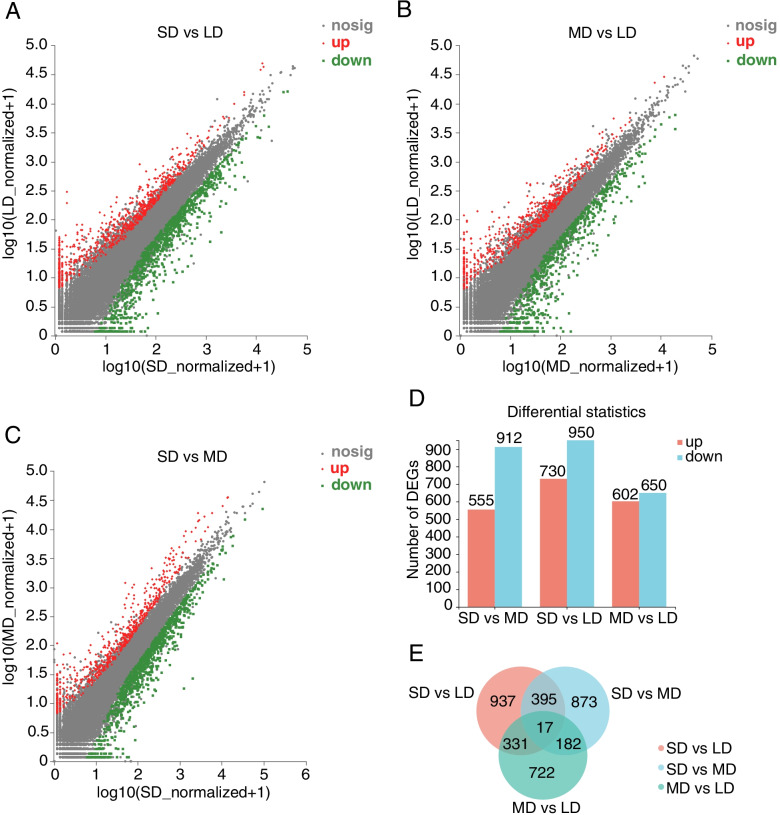
The graphical representation of differentially expressed genes (DEGs) of *C. oleifera* ‘Huanshuo’ in response to different photoperiod. The scatter plot of all DEGs in the SD_MD (**A**), MD_LD (**B**), and SD_LD (**C**) differential expression gene sets; (**D**) Number of up/down-regulated DEGs in the SD_MD, SD_LD, and MD_LD differential expression gene sets; (**E**) Venn diagram of all DEGs numbers among the three gene sets

We used the reads per kilobase of transcript per million reads mapped (RPKM) method to measure gene expression levels. Scatter plots were used to illustrate all DEGs in the SD_LD, MD_LD, SD_MD, and differential expression gene sets (Fig. 2A-C). We observed the lowest number of DEGs between the MD and LD groups, where the MD group had 602 upregulated genes and 650 downregulated genes. We observed the highest number of DEGs between the SD and LD groups, where the SD group had 730 upregulated genes and 950 downregulated genes. The total number of DEGs between the SD and MD groups was 1467, where the SD group had 555 upregulated genes and 912 downregulated genes (Fig. 2D). We used Venn diagrams to summarize the counts of DEGs between all combinations of the SD, MD, and LD groups. Moreover, 17 DEGs were shared among all sets (Fig. 2E).

**Fig. 3:**
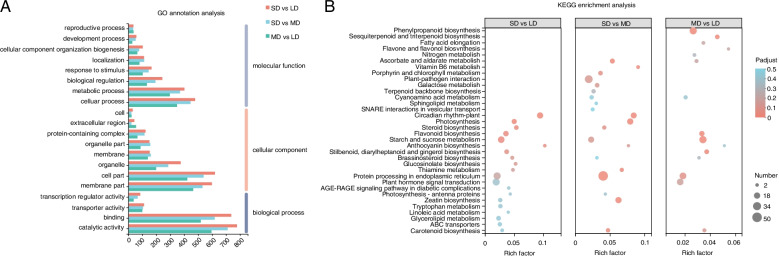
Functional annotation of the assembled transcriptome in SD_MD, MD_LD, and SD_LD differential expression gene sets. GO classification analysis (**A**) and KEGG enrichment analysis (**B**) of SD_MD, MD_LD, and SD_LD differential expression gene sets

From GO enrichment analysis, we found that DEGs enriched in the “cellular process” and “metabolic process” terms of the GO “molecular function” categories. In the “cellular component” GO category, DEGs enriched in the “membrane part” and “cell part” terms. In the “biological process” GO category, DEGs enriched in the “binding” and “catalytic activity” terms (Fig. 3A). We used KEGG enrichment analysis to illustrate the enrichment results of the top 20 DEGs, which showed significant enrichment in many terms related to signal transduction pathways, including “plant hormone signal transduction,” “protein processing in endoplasmic reticulum,” “phenylpropanoid biosynthesis,” and “starch and sucrose metabolism” (Fig. 3B). These results provide important insight into the photoperiodic adaptation mechanism of *C. oleifera*.

### Weighted gene co-expression network analysis (WGCNA) of genes related to photoperiod sensitivity in *C. oleifera*

#### Construction of co-expression gene networks

**Fig. 4:**
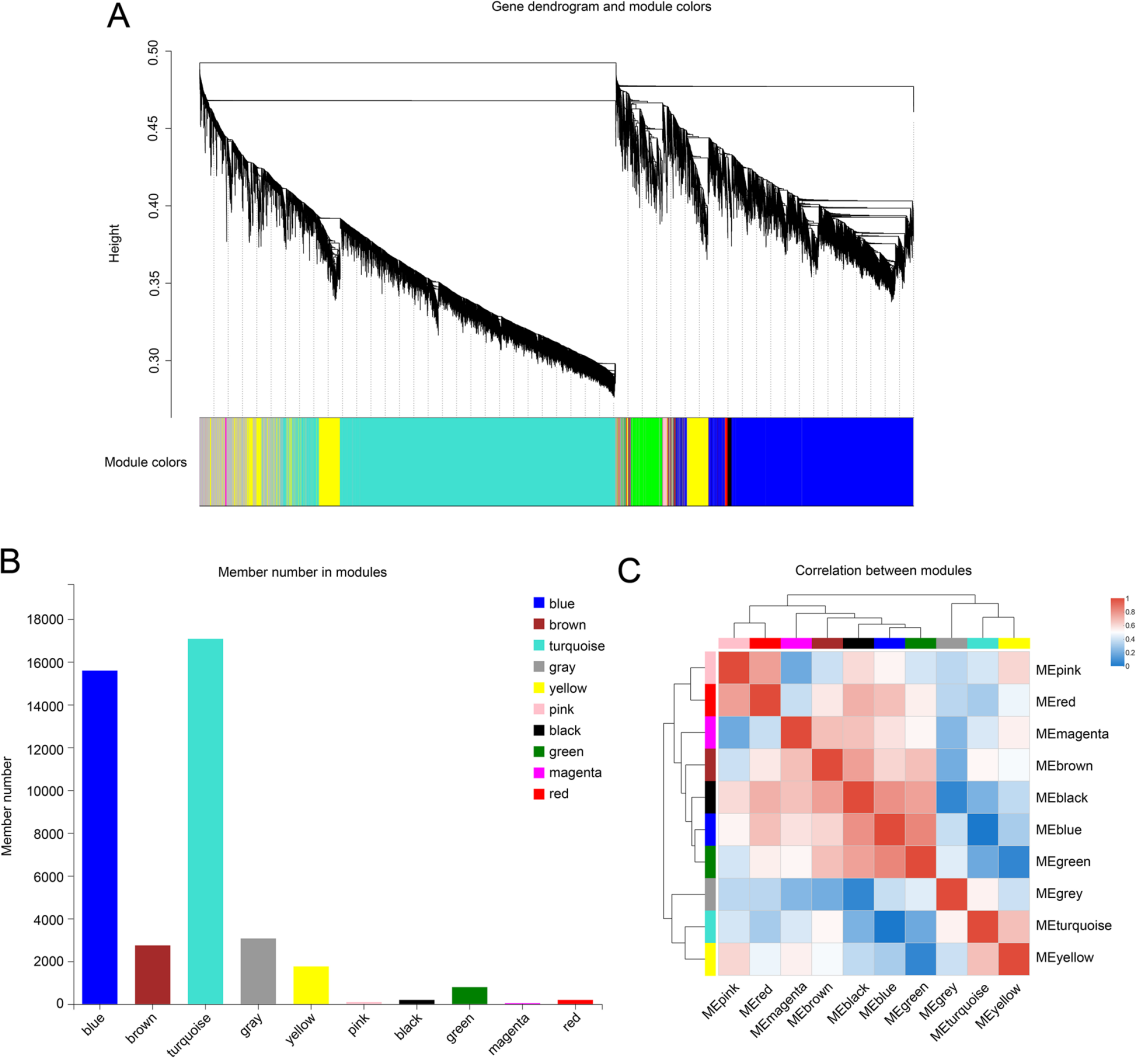
WGCNA of genes related to photoperiod of *C. oleifera*. **A** Clustering dendrograms of genes. Dissimilarity was based on topological overlap, together with assigned module colors. The 10 coexpression modules are displayed in different colors. **B** Member number in 10 modules. **C** Correlation between 10 modules

We used WGCNA to further investigate the genes of *C. oleifera* involved in photoperiod sensitivity (Fig. 4A). After background correction and standardization of 110,384 transcripts, we filtered genes with small or abnormal variations. The intensity of correlation between the 41,514 treated genes was consistent with a scale-free distribution (Fig. S4). Ten gene modules were identified, which were labeled using different colors (Figs. 4B, C and S5). The gene counts of the ten modules ranged from 42 to 17,068 (Fig. 4B).

#### Identification of modules related to SD, MD, and LD

**Fig. 5:**
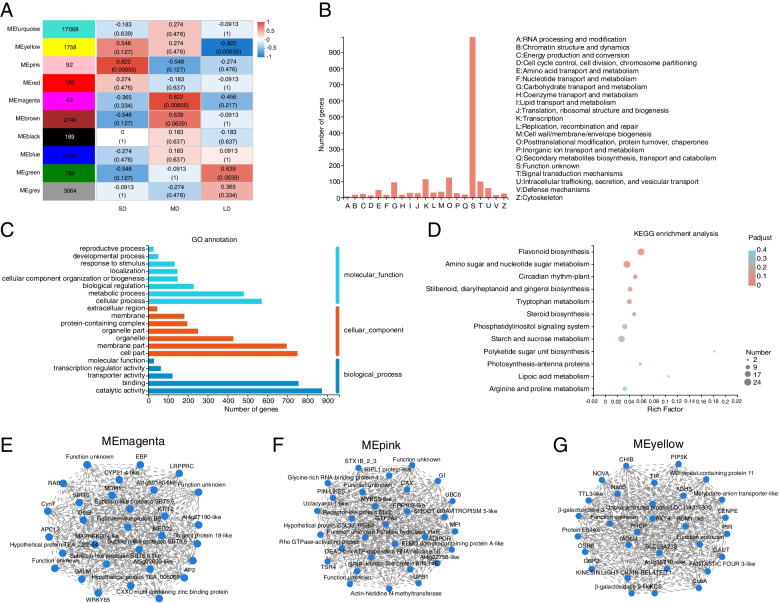
Module-trait associations and visual analysis. **A** Correlation coefficients between different modules and traits show by matrix. COG annotations (**B**), GO classification analysis (**C**) and KEGG enrichment analysis (**D**) of MEmagenta, MEpink and MEyellow modules. Visual analysis of MEmagenta (**E**), MEpink (**F**) and MEyellow (**G**) modules

The variation in gene expression between the SD, MD, and LD groups was relatively high, which suggests that photoperiod has an important effect on flowering in *C. oleifera*. We paid particular attention to the gene expression in three modules that were significantly correlated with photoperiod (*P* < 0.05): Genes in pink module (92 genes) were positively correlated with SD, and negatively correlated with both MD and LD. Genes in the magenta module (42 genes) were positively correlated with MD and negatively correlated with both LD and SD. Finally, genes in the yellow module (1758 genes) were positively correlated with both SD and MD, but negatively correlated with LD (Fig. 5A).

#### Functional specific enrichment analysis of related modules

Our analysis using the COG database revealed that genes in the pink, magenta, and yellow modules enriched in the categories posttranslational modification, protein turnover, chaperones, transport, carbohydrate transport and metabolism (Fig. 5B). Furthermore, GO enrichment analysis of the pink, magenta, and yellow modules revealed that DEGs enriched in the “cellular process” term of the “molecular function” category. In the “cellular component” GO category, DEGs enriched in the “cell part” term. In the “biological process” category, DEGs enriched in the “catalytic activity” term (Fig. 5C). From KEGG enrichment analysis of the three modules, we found that genes associated with the “flavonoid biosynthesis,” “amino sugar and nucleotide sugar metabolism,” and “circadian rhythm-plant” terms were enriched (Fig. 5D).

#### Identification of hub genes

Hub genes, which have a high degree of co-relationships and connectivity among modules, were identified using by visualization network analysis [25]. Analysis was conducted on the top 30 nodes of connectivity in the pink, magenta, and yellow modules, as well as on connections between nodes with weights greater than 0.02 (Fig. 5E-G). From our results, we focused on the following three hub genes: *WRKY65* and *AP2* in the magenta module, and *GI* in the pink module. As previously reported, *GI* genes control circadian rhythm and photoperiodic flowering in *Arabidopsis* [26, 27], and the *AP2-like* gene plays a key role in the photoperiodic-based flowering induction of *Ipomoea nil* [28]. Furthermore, *WRKY65* expression has been reported to be affected by FLD, the key factor present in the flowering mechanism [29]. Therefore, the hub genes *WRKY65*, *AP2*, and *GI* may play important roles in the photoperiodic flowering of *C. oleifera*.

#### Transcription factor (TF) prediction and analysis in the related modules

**Fig. 6:**
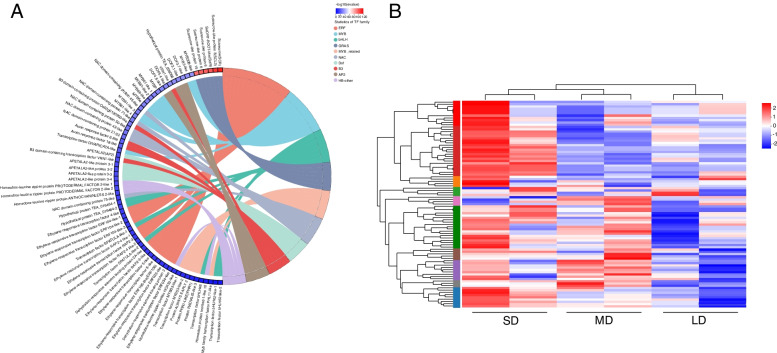
Transcription factor (TF) family statistics and heatmap analysis in MEmagenta, MEpink and MEyellow modules. **A** TF prediction and family statistics of three modules. **B** Heatmap of TFs in three modules. Red and Blue represents up- and down-regulated DEGs, respectively

We aligned the putative protein sequences to the Plant TFdb database for TF prediction. As shown in Fig. S6, a total of 5856 expressed TFs belonging to 48 TF families were identified from the transcriptome analysis of *C. oleifera*. TFs play an important role in the photoperiodic flowering pathway. For example, the TF ZmNF-YA3 has been found to promote photoperiod-dependent flowering in maize by interacting with FPF1 and CO-like proteins to form a heterotrimeric complex, which subsequently binds to the promoter of *ZmFTlike12* [30]. Our WGCNA results showed that gene expression in the three aforementioned modules was significantly correlated with the photoperiod. We identified 99 TFs belonging to 27 TF families in these three modules (Fig. 6). The ten most abundant TF families were *ERF*, *MYB*, *bHLH*, *GRAS*, *MYB*_related, *NAC*, *Dof*, *B3*, *AP2* and *HB*-other. Our results suggest that the genes in three modules may be pivotal regulators in the photoperiod flowering process of *C. oleifera*, but this finding requires further verification.

### Network analysis of the hub genes

**Fig. 7:**
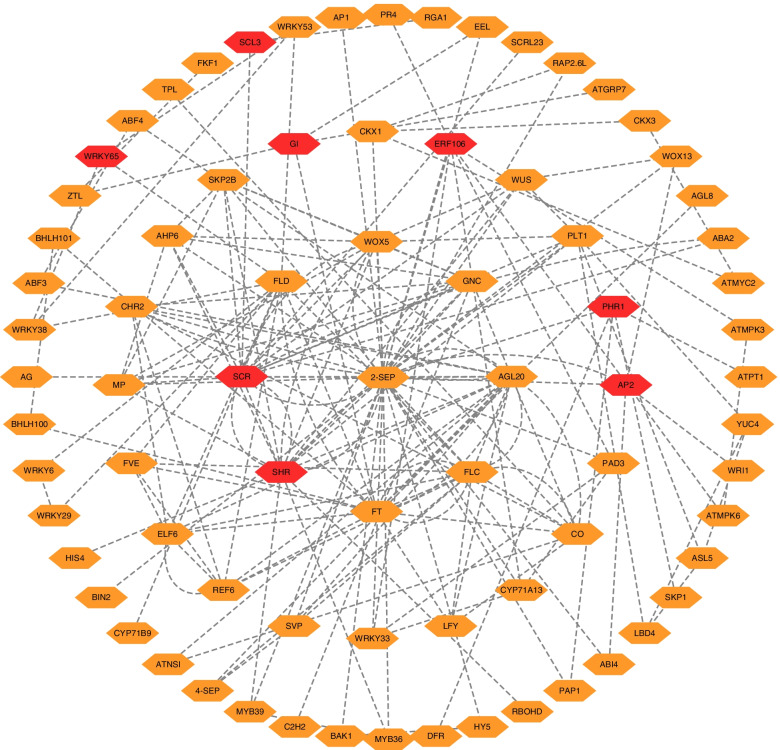
Construction of Hub genes and TFs regulation networks about photoperiod flower pathway by Cytoscape software. Red and yellow hexagon represent identified hub genes involved in the network

We performed network analysis to investigate the interaction between hub genes and the TFs involved in the flowering pathways. Cytoscape software was used to construct a regulation network of the hub genes and TFs, which had 77 nodes, 181 edges, and eight hub genes (*GI*, *AP2*, *WRKY65*, *SCR*, *SHR*, *PHR1*, *ERF106*, and *SCL3*) (Fig. 7). Our Network analysis showed that *PHR1*, *SCR*, *ERF106*, and *SHR* directly interact with *2-SEP*, and further interact with *FT*, *AGL20*, *LFY*, and *CO* to function within the photoperiodic flowering pathway. Meanwhile, we also found that *WRKY65* interacts with certain targets, such as *WRKY53*, *WRKY38*, and *FLD*, and then interacts with *FT*, and *FLC* to function within this pathway. It is worth noting that *WRKY65* expression may be affected by *FLD* function in systemic acquired resistance [29]. We also found that GI may interact with *ZTL*, *FT*, *EEL*, and *ATGRP7*, and that *AP2* may interact with *WOX13*, *WRI1*, *2-SEP*, and *ABI4*, to regulate the pathway. Ultimately, these results suggest that three hub genes (*GI*, *AP2*, and *WRKY65*) and five TFs (*SCR*, *SHR*, *PHR1*, *ERF106*, and *SCL3*) may play important roles in the photoperiodic flowering mechanism of *C. oleifera*. Furthermore, *2-SEP*, *FT*, *CO*, *AGL20* and *FLC* may be key nodes for other hub genes and TF regulation related to this pathway.

### qRT-PCR analysis of the hub genes

**Fig. 8:**
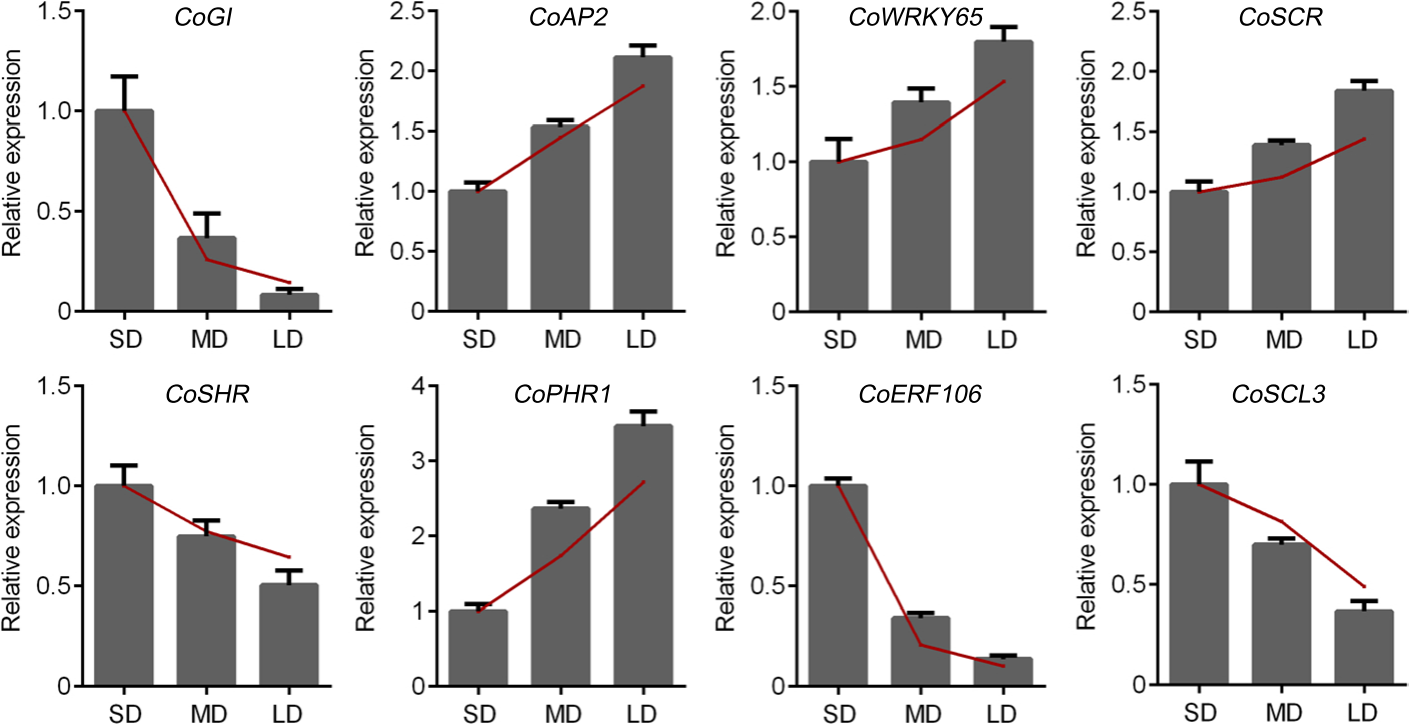
qRT-PCR analysis of hub genes and TFs (*CoGI*, *CoAP2*, *CoWRKY65*, *CoSCR*, *CoSHR*, *CoPHR1*, *CoERF106* and *CoSCL3*) about photoperiod flower pathway

To validate our RNA-Seq results, we performed qRT-PCR to measure the expression levels of the eight hub genes and the TFs. Our results showed that the expression levels of *CoGI*, *CoSHR*, *CoERF106*, and *CoSCL3* were increased in the SD group relative to the MD group. Additionally, the expression levels of these genes were all increased in the MD group relative to the LD group. The expression levels of *CoAP2*, *CoWRKY65*, *CoSCR*, and *CoPHR1* showed the opposite pattern (Fig. 8). Overall, our results suggest that these eight hub genes function within the photoperiodic flowering pathway of *C. oleifera*.

## Discussion

*C. oleifera* is an important woody oil tree species in China that can produce high quality edible oil. Flowering is the transition of higher plants from the vegetative growth stage to the reproductive growth stage. Flower formation and flowering time play key roles in the oil production of *C. oleifera*. From our phenotype data, we found that photoperiod affects flowering time in *C. oleifera* – more specifically, the longer the duration of light, the earlier the flowering time. The phenotypic changes that we observed were similar to those of *Arabidopsis* [31], wheat [32], and rapeseed [33]. The content levels of soluble protein and soluble sugar typically change with the flowering process. For example, different temperatures, photoperiods, and spermidine concentrations can regulate soluble sugar and soluble protein contents to control flowering time in *Anoectochilus roxburghii* [34]. As previously reported, sugar levels delay seed germination and stimulate the induction of both flowering and senescence in some plant species [35]. In our study, the soluble protein and soluble sugar contents in *C. oleifera* subjected to different light conditions ranked in the following order: LD > MD > SD. This finding was consistent with the flowering phenotypes we observed in our study. Whereas previous studies have mainly focused on the cultivation and breeding of *C. oleifera* [36–38], our study adds to collective knowledge on the plant’s regulation of flowering stage.

Although the genome of *C. oleifera* has previously been reported [39, 40], the species’ large genome, polyploidization, and frequent interspecific hybridization complicate genome assembly and gene function analysis. Alternatively, RNA-Seq is an effective technique that can be used to explore and analyze genetic characteristics. Based on phenotypic and physiological data, transcript profiling of *C. oleifera* grown in different photoperiod conditions (SD, MD, and LD) provided useful information for understanding the molecular mechanism that affects photoperiodic flowering, thus improving our ability to cultivate new strains of *C. oleifera* breeding. Overall, our genome-wide transcriptomic and gene network analyses of *C. oleifera* subjected to different photoperiod conditions revealed the functional and regulatory genes involved in the plant’s photoperiodic-responsiveness.

We obtained transcriptome information by performing RNA-Seq on the mature leaves of *C. oleifera* grown under different photoperiod conditions (SD, MD, and LD). We identified 1207 genes with significant differential expression between the MD and LD groups, 1680 genes between the SD and LD groups, and 1467 genes between the SD and MD groups. These results indicate that many genes are involved in regulating the photoperiodic flowering process of *C. oleifera*. KEGG enrichment analysis of the top 20 DEGs showed significant enrichment within the pathways of starch and sucrose metabolism and protein processing in the endoplasmic reticulum. Additionally, the results of our transcriptome analysis were consistent with the soluble protein and soluble sugar contents we observed (Fig. 1C, D). Overall, these results help us gain insight into the metabolic processes involved in the photoperiodic response of *C. oleifera*.

We used WGCNA to explore gene association patterns and evaluate the potential interactions between expressed genes. As shown by our results, the regulatory networks and large gene clusters significantly differed between samples grown in the SD, MD, and LD conditions. Moreover, our study identified three modules with a high correlation to photoperiod. Genes in the pink module (92 genes) were upregulated in the SD group, genes in the magenta module (42 genes) were upregulated in the MD group, and genes in the yellow module (1758 genes) modules were down-regulated in the LD group (Fig. 6). KEGG analysis of the three modules identified flavonoid biosynthesis, amino sugar and nucleotide sugar metabolism, and circadian rhythm-plant as the top three enrichment pathways. Flavonoid biosynthesis is known to impact the flower development, and especially flower color [41, 42]. As previously reported, different light intensities and circadian rhythms can affect flavonoid biosynthesis in plants [43, 44]. According to our results, flavonoid biosynthesis may impact the flowering time of *C. oleifera* in response to different photoperiod conditions. The circadian clock in leaves can convert exogenous photoperiod signals into endogenous signals to initiate floral transition [45]. Other studies have shown that a series of feedback circuits form the core clock in *Arabidopsis*, whose components reciprocally or sequentially repress one another, and include a set of key genes (*CCA1*, *TOC1*, and *PRR1*) [46–48]. Our results indicate that circadian rhythm plays an important role in the photoperiodic signaling of *C. oleifera*.

In our study, the results of WGCNA identified eight hub genes (*GI*, *AP2*, *WRKY65*, *SCR*, *SHR*, *PHR1*, *ERF106*, and *SCL3*). The same trends in these genes were observed between the results of qRT-PCR and RPKM, suggesting that the expression of hub genes corresponded well between the two methods. GI, a plant specific nuclear protein, functions in diverse physiological processes, including flowering time regulation and the control of circadian rhythm in plants [49, 50]. Our results identified the photoperiod related gene *GI* as a hub gene, which demonstrates the high reliability of our transcriptomic analysis. AP2, a floral homeotic transcription factor belonging to the AP2/EREBP (ethylene responsive element binding protein) class, is involved in the specification of floral organ identity, suppression of floral meristem indeterminacy, establishment of floral meristem identity, and development of ovule and seed coat [51]. Meanwhile, both basal and SAR-induced expression of *WRKY65* are regulated by FLD, the key factor of flowering [29]. Additionally, photoperiod flowering pathways are regulated by gibberellin (GA) signaling pathways [52–55]. Our WGCNA results suggest that photoperiodic flowering involves a complex regulatory network related to GA signaling, which contains the GRAS family SCR, SHR, and SCL3. As previously reported, ethylene-responsive transcription factor ERF106 mediates Na+/H + transport to enhance salt tolerance in apples [56]. The transcription factor PHR1 contributes to the homeostasis of both sulfate and phosphate in plants, enhances the adaptation of plants to high levels of light, and maintains functional photosynthesis to avoid permanent damage [57]. However, few reports exist on the functions of *ERF106* and *PHR1* within the photoperiodic flowering pathway. Our study identified eight hub genes that may play key roles in this pathway – an enhanced understanding of the function of these genes requires further study. Furthermore, the results of qRT-PCR verified the accuracy of our transcriptomic analysis.

## Conclusion

To explore the sensitivity of *C. oleifera* to different photoperiods, we determined the flowering time of *C. oleifera* grown under SD, MD, and LD conditions. Our phenotypic analysis showed that the different photoperiods significantly impacted flowering time. Transcriptome analysis was performed to investigate the photoperiodic flowering mechanism of *C. oleifera*. Our results showed that the “flavonoid biosynthesis,” “amino sugar and nucleotide sugar metabolism,” and “circadian rhythm-plant” pathways play key roles in this mechanism. We also identified eight hub genes (*GI*, *AP2*, *WRKY65*, *SCR*, *SHR*, *PHR1*, *ERF106*, and *SCL3*) that may be involved in this mechanism. Overall, our RNA-seq data provides valuable resources and sequences for investigating the genetic basis of photoperiodic sensitivity in *C. oleifera*.

## Materials and methods

### Plant material and growth conditions

On October 20, 2020, the 4-year-old *C. oleifera* cultivar ‘Huashuo’, which grew well without disease and with similar growth potential, were planted in the greenhouse of the Central South Forestry University of Science and Technology located in Changsha, China (28°10’ N, 113°23’ E). The same fertilizer and water managements were conducted for plants. On January 4, 2022, sixty four-year-old C. *oleifera* ‘Huashuo’ were divided into SD (8 h light/16 h dark), MD (12 h light/12 h dark) and LD (16 h light/8 h dark) groups at random in the greenhouse.

### Determination of Physiological Indexes of *C. oleifera* ‘Huashuo’

#### Method for determination of soluble protein content

According to the method Braford [58], the Coomassie Brilliant Blue G-250 method was used to determine soluble protein content. 0.2 g *C. oleifera* leaves in liquid nitrogen were grounded to power, and 6 ml PBS (0.05 mol/L, PH7.0) which were pre-cooled at 4 ℃ add to the power, and the suspension centrifuged at 4 ℃ for 10 min. The supernatant was protein extract. 0.6 ml extraction added 5 ml Coomassie Brilliant Blue G-250 solution and 1.4 ml PBS, fully shocked for 5 min. A595 was measured. The soluble protein content was calculated according to the standard curve.

#### Method for determination of soluble sugar content

According to the method of Tang [59], 0.2 g *C. oleifera* leaves in liquid nitrogen were grounded into powder, and put into test tubes with containing 20 ml ddH_2_O and extracted in a 100 ℃ water bath for 0.5 h. After filtration, the supernatant was added up to 100 ml by addition of ddH_2_O. Then a mixture containing 6.5 ml anthrone ethyl acetate reagent, 1 ml sample and 1.5 ml ddH_2_O, and was shaken and heated for 10 min. To measure soluble sugar content, the A620 was recorded and the soluble sugar content was calculated according to the standard curve.

### Sampling for RNA-Seq

During pre-flowering period, the same part mature leaves of *C. oleifera* were collected for molecular sequencing analyses in different photoperiod. Three biological repeat samples were selected for each treatment and named SD-1, SD-2, SD-3, MD-1, MD-2, MD-3, LD-1, LD-2, and LD-3, respectively. All samples were rapidly frozen in liquid nitrogen and stored at -80 ℃. Nine samples were used for the RNA-Seq analyses. TRIzol reagent (Life Technologies) was used to extract total RNA and processed following the protocol provided by the manufacturer. Nanodrop2000 (ThermoFisher) was used for purity and concentration detection of the extracted RNA, RNA integrity was detected by agarose gel electrophoresis, and Agilent 2100 was used to determine the RIN value. Nine RNA samples were sent to Shanghai Meiji Biomedical Technology Co., Ltd. (Pudong New Area, shanghai, China) for library preparation and RNA sequencing. According to Illumina TruseqTM RNA sample prep Kit method, 1 µg of equally mixed RNA was extracted from 9 samples to prepare library. Illumina sequencing platform was used for RNA sequencing. Sequencing data quality control includes sequencing data statistics. Original data statistics and quality control data statistics.

### Transcriptome assembly and gene functional annotation

Software Cufflinks (http://cole-trapnelllab.github.io/cufflinks/) [60] or StringTie (http://ccb.jhu.edu/software/stringtie/) [61] was used to assemble reads together, then compared with the known transcription, got transcript annotation information and the potential new transcript for functional annotation. Moreover, gene function was annotated based on Gene Ontology (GO), the Kyoto Encyclopedia of Genes and Genomes (KEGG) [62], Clusters of Genes (COG), NCBI non-redundant protein sequences (NR), a manually annotated and reviewed protein sequence database (Swiss-prot), and Protein family (Pfam) databases.

### Differential expression analyses

RPKM was used to estimate gene expression levels [63]. The fragments per kilobase of transcript per million mapped reads (FPKM) method can eliminate the influence of differences in sequencing amount and gene length on the calculation of gene expression. DESeq2 was used for differential expression analyses of two sample groups at different photoperiod conditions [64]. The BH (FDR correction with Benjamini/Hochberg) method was used to perform multiple test corrections and the *p*-value after correction is p-adjust. P-adjust < 0.05 and |log2FC| >=1 was used as the screening indexes for significantly differentially expressed genes.

### Transcription factor analysis

The PlantTFDB database was used for transcription factor prediction. Moreover, controls option was set by Blast *E*-value (1.0E-5) and choosing Hummsacn *E*-value (1.0E-5).

### Construction of gene co-expression networks

Weighted gene co-expression network analysis (WGCNA) R package (version 3.6) [25] was used to conduct co-expression networks and module detection. The parameter of signed network type, soft power of 1, 30 minModulesize, 0.3 minKMEtoStay, 0.25 mergeCutHeight were selected for module recognition. The associations between trait and modules were estimated using spearman correlation coefficient between phenotype and module eigengenes.

### Network analysis of hub genes

Cytoscape-V3.9.0 with the Agilent Literature Search Plug-in was used to construct and analyze the network [65]. Control options of search by using the default parameters, including selection of ‘concept lexicon restricts’ and ‘use context’, max engine matches of 10. Meanwhile, *Arabidopsis thaliana* in concept lexicon, and choosing ‘relaxed’ in interaction lexicon were set for extraction controls option. Attribute circle layout was used to conduct network.

### qRT-PCR analysis

Total RNA was extracted by using the EZ-10 DNAaway RNA small amount extraction kit (Sangon Biotech), and reverse transcribed using hiscript III 1st strand cDNA synthesis Kit (+ gDNA wiper) kit (Vazyme). The expression of *CoGAPDH* was used as internal control. The primers used are listed in Table S2. Each data point represents the average of three biological replicates.

## Supplementary Information

**Additional file 1.**

## Data Availability

The raw sequence reads were deposited into NCBI SRA database under accession no. PRJNA798595 (https://www.ncbi.nlm.nih.gov/bioproject/PRJNA798595).
