# Supplementary material for: Analysis of Camellia oleifera transcriptome reveals key pathways and hub genes involved during different photoperiods
Source: BMC Plant Biol. 2022 Sep 12;22:435. doi: 10.1186/s12870-022-03798-0 (PMC9465947; doi:10.1186/s12870-022-03798-0)
Supplement: Supplementary file 1 — Additional file 1. [file 12870_2022_3798_MOESM1_ESM.docx]

**
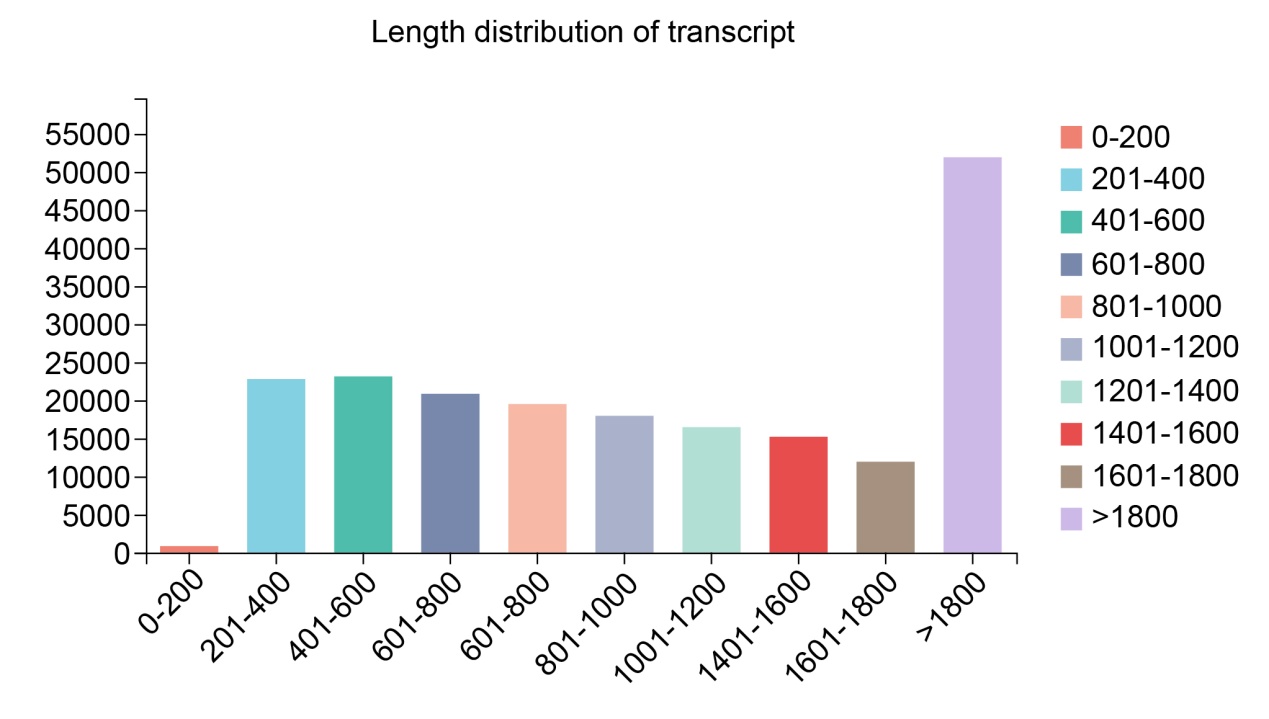
**

**Fig.S1** Length distribution of transcripts.

**
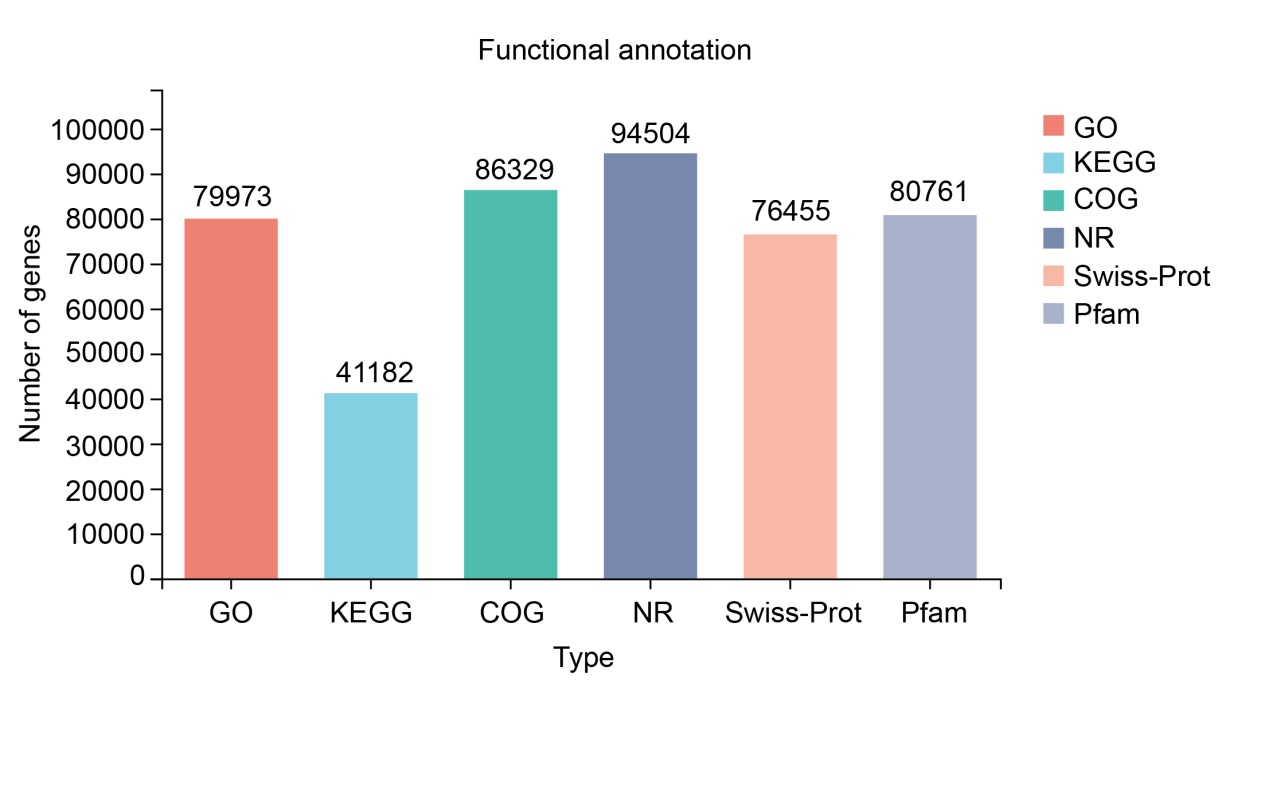
**

**Fig.S2** Annotation of the *C. oleifera* ‘Huashuo’ transcriptome to GO, COG, Pfam, Swiss-Prot, NR and KEGG database.

**
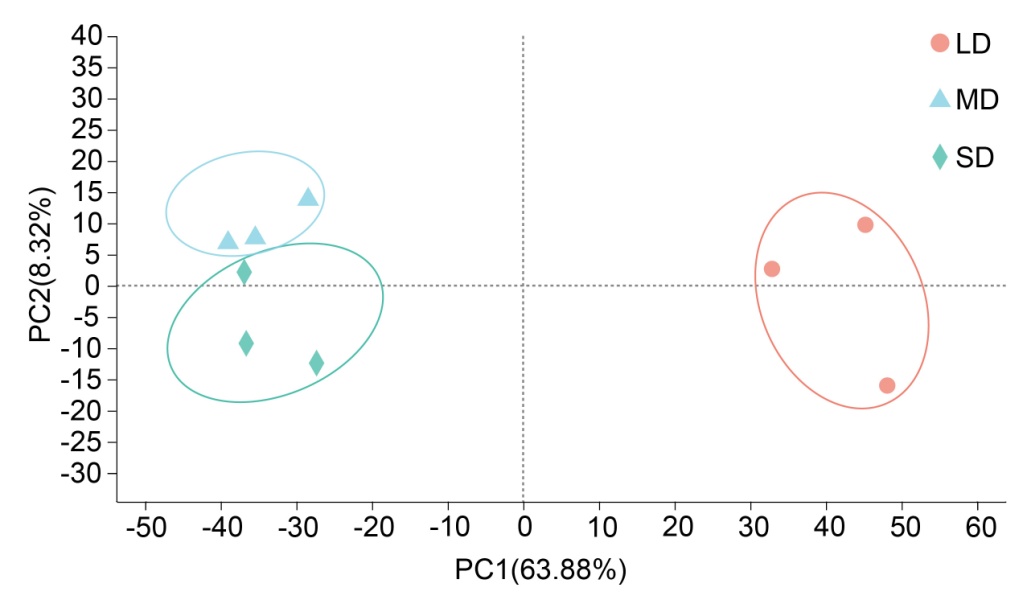
**

**Fig.S3** Principle component analysis (PCA) of nine samples.

**Table.S1** Overview of the sequencing and assembly

| Sample  ID | Raw  reads | Raw  bases | Clean reads | Clean  bases | GC  content (%) | Error rate  (%) | Q30  (%) |
| --- | --- | --- | --- | --- | --- | --- | --- |
| SD_1 | 54117574 | 8171753674 | 53378550 | 7813905744 | 46.48 | 0.0252 | 93.93 |
| SD_2 | 46049218 | 6953431918 | 45660408 | 6729746592 | 45.11 | 0.0253 | 93.85 |
| SD_3 | 61883056 | 9344341456 | 61333744 | 9025259028 | 45.29 | 0.0254 | 93.8 |
| MD_1 | 48941470 | 7390161970 | 48479022 | 7108284387 | 44.89 | 0.0256 | 93.6 |
| MD_2 | 49408366 | 7460663266 | 48932002 | 7120322373 | 45.11 | 0.0252 | 93.98 |
| MD_3 | 51142238 | 7722477938 | 50598180 | 7450714406 | 44.94 | 0.0254 | 93.79 |
| LD_1 | 49855982 | 7528253282 | 49194450 | 7254389655 | 45.8 | 0.0254 | 93.73 |
| LD_2 | 47475124 | 7168743724 | 46972220 | 6921724082 | 45.06 | 0.0259 | 93.25 |
| LD_3 | 51482826 | 7773906726 | 50976180 | 7516889944 | 44.99 | 0.0253 | 93.89 |


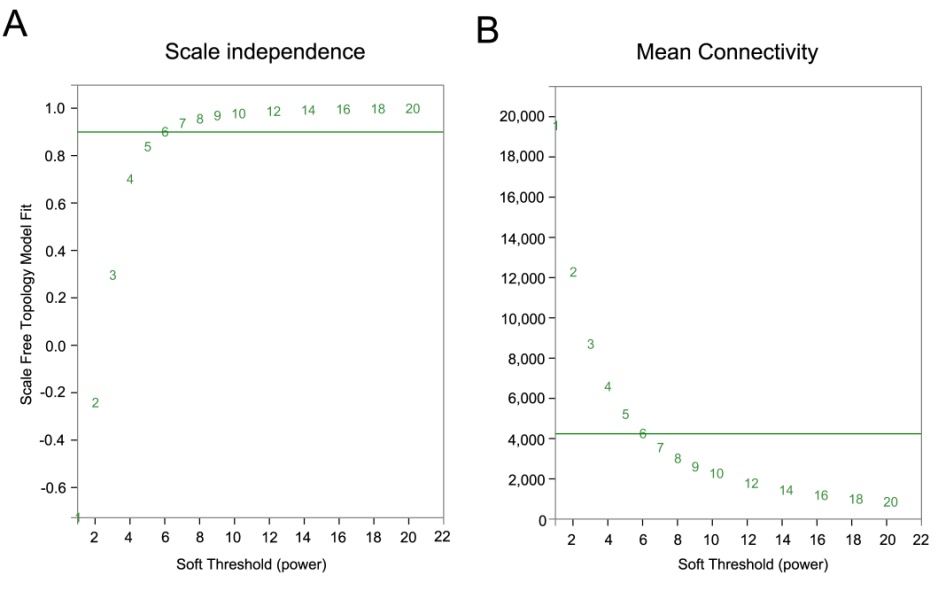


**Fig.S4** Determination of power β-value based on the adjacency matrix using WGCNA. The adjacency matrix from co-expression data was weighted by the power of correlation data between diferent genes. Scale free topology model fit (**A**) and mean connectivity (**B**) were conducted to ensure that the average connectivity of the network was smooth.


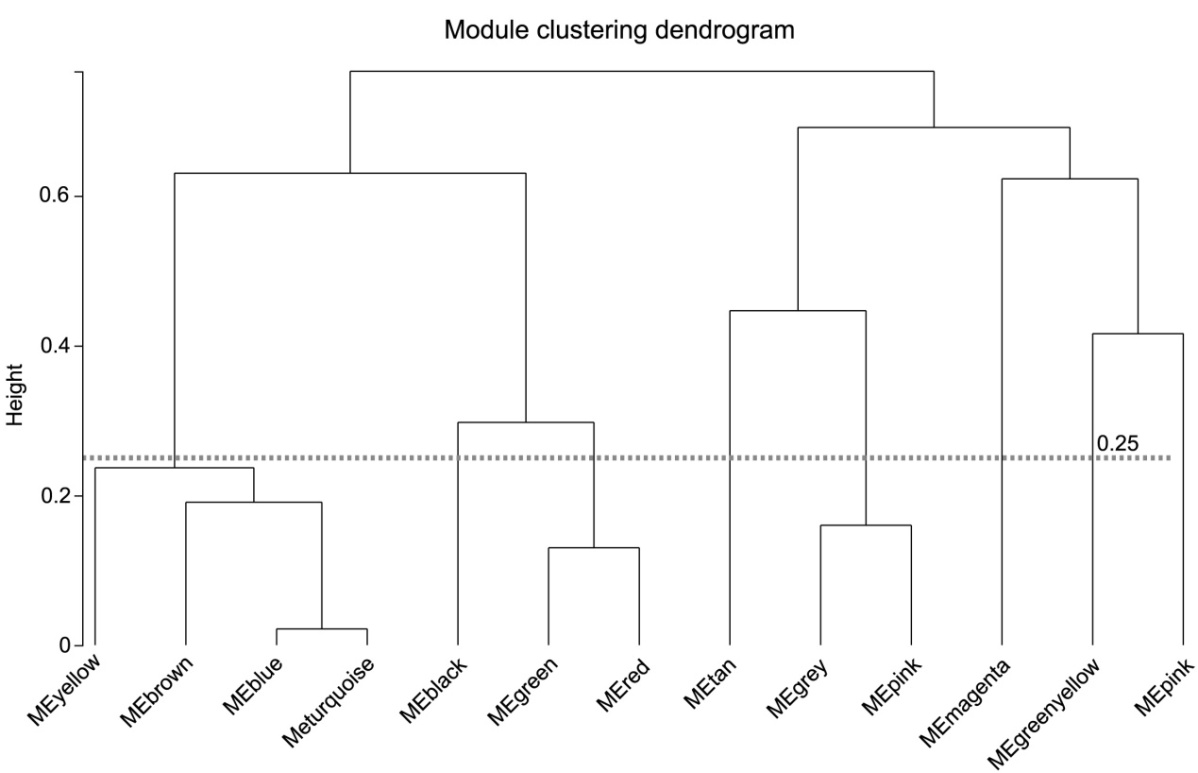


**Fig.S5** Module cluster analysis. The branch represents a module and the ordinate is the clustering distance.


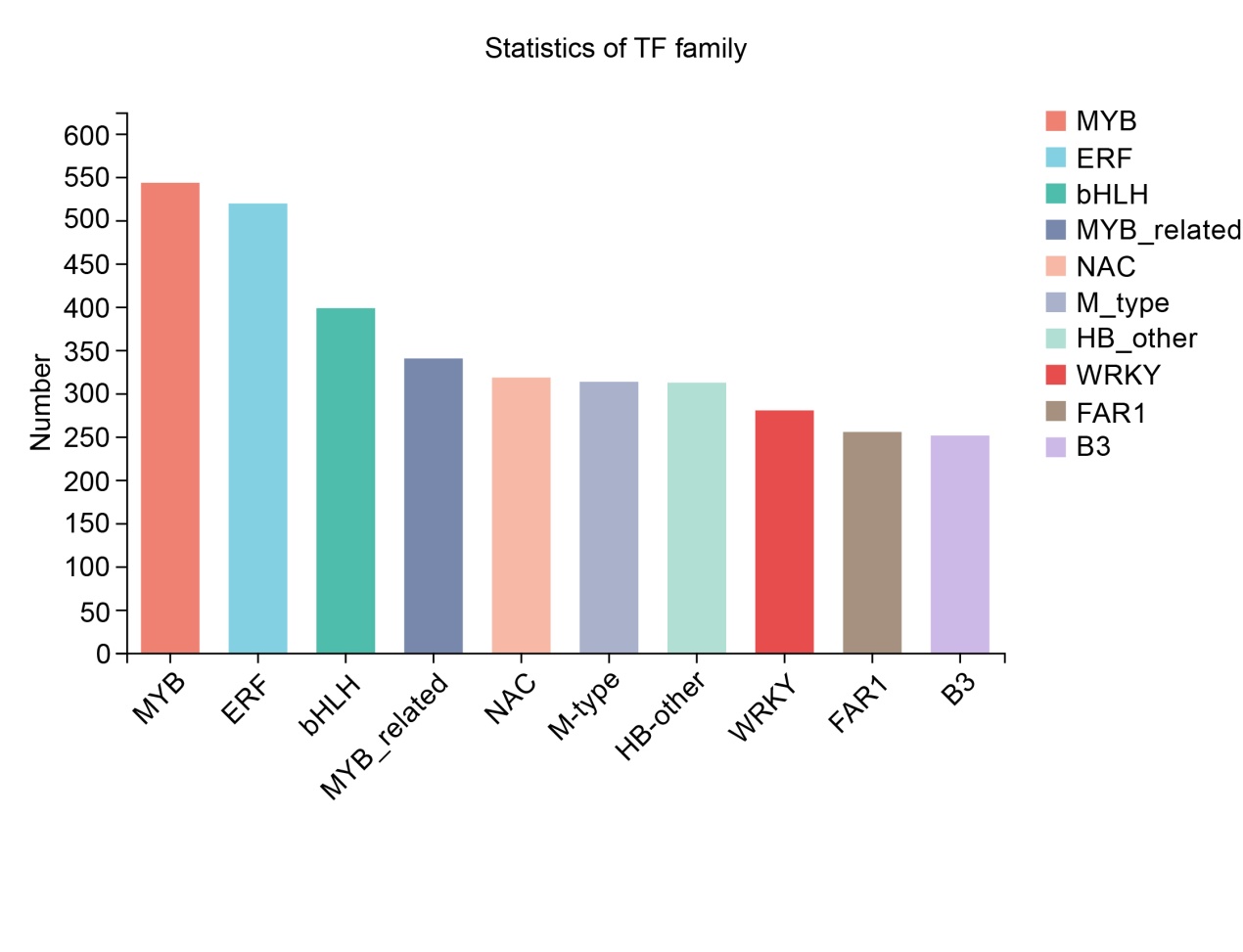
**Fig.S6** TF prediction and statistics of TF family.

**Table S2.** Primer sequences used in this study.

| **Destination**  **products** | **Primer**  **name** | **Primer sequence** |
| --- | --- | --- |
| *CoGAPDH* | *CoGAPDH*-QF  *CoGAPDH*-QR | 5’- CTACTGGAGTTTTCACCGA -3’  5’- TAAGACCCTCAACAATGCC -3’ |
| *CoGI* | *CoGI*-QF  *CoGI*-QR | 5’-TATGTATTAGCGGCTGTCTGTG -3’  5’-CATTTGAACTGTAACTCCACGAC -3’ |
| *CoWRKY65* | *CoWRKY65*-QF  *CoWRKY65*-QR | 5’- ACCATCATCTCCGCCACT -3’  5’- CACCGAACAACGACTCATCC -3’ |
| *CoAP2* | *CoAP2*-QF  *CoAP2*-QR | 5’- GACGCCGACGAGGATAAG -3’  5’- CTGACGGAGTGCCCATTT -3’ |
| *CoSCR* | *CoSCR*-QF  *CoSCR*-QR | 5’- AATACCCGCCTTCTCCTGAT -3’  5’- CCCCTTCCTAAACTGCCAAA -3’ |
| *CoSHR* | *CoSHR*-QF  *CoSHR*-QR | 5’- GAGCCCTTGGACTACCTTCG -3’  5’- CACCCATGAGTCTAGCGAAC -3’ |
| *CoPHR1* | *CoPHR1*-QF  *CoPHR1*-QR | 5’- CCCGATAAAGCAACACCTAA -3’  5’- GTACTCGCAAAGCCTCAGTAA -3’ |
| *CoERF106* | *CoERF106-*QF  *CoERF106-*QR | 5’- ACTCATACGCCAACACCTCC -3’  5’- TCCGCCTCATCACCATTC -3’ |
| *CoSCL3* | *CoSCL3-*QF  *CoSCL3-*QR | 5’- GTGGTTCTGGCGGTGAGTT -3’  5’- TTCGGCAAAGGATTGGTCT -3’ |
